# Diversity, Differentiation, and Linkage Disequilibrium: Prospects for Association Mapping in the Malaria Vector *Anopheles arabiensis*

**DOI:** 10.1534/g3.113.008326

**Published:** 2013-11-26

**Authors:** Clare Diana Marsden, Yoosook Lee, Katharina Kreppel, Allison Weakley, Anthony Cornel, Heather M. Ferguson, Eleazar Eskin, Gregory C. Lanzaro

**Affiliations:** * Vector Genetics Laboratory, Department of Pathology, Microbiology, and Immunology, School of Veterinary Medicine, University of California-Davis, California 95616; † Ifakara Health Institute, Off Mlabani Passage, Ifakara, United Republic of Tanzania; ‡ Boyd Orr Centre for Population and Ecosystem Health, University of Glasgow, Glasgow, G12 8QQ, UK; § Department of Entomology, University of California-Davis, California 95616; ** Department of Computer Science, University of California Los Angeles, California 90095

**Keywords:** *Anopheles arabiensis*, linkage disequilibrium, association mapping, malaria vector, inversion

## Abstract

Association mapping is a widely applied method for elucidating the genetic basis of phenotypic traits. However, factors such as linkage disequilibrium and levels of genetic diversity influence the power and resolution of this approach. Moreover, the presence of population subdivision among samples can result in spurious associations if not accounted for. As such, it is useful to have a detailed understanding of these factors before conducting association mapping experiments. Here we conducted whole-genome sequencing on 24 specimens of the malaria mosquito vector, *Anopheles arabiensis*, to further understanding of patterns of genetic diversity, population subdivision and linkage disequilibrium in this species. We found high levels of genetic diversity within the *An. arabiensis* genome, with ~800,000 high-confidence, single- nucleotide polymorphisms detected. However, levels of nucleotide diversity varied significantly both within and between chromosomes. We observed lower diversity on the X chromosome, within some inversions, and near centromeres. Population structure was absent at the local scale (Kilombero Valley, Tanzania) but detected between distant populations (Cameroon *vs.* Tanzania) where differentiation was largely restricted to certain autosomal chromosomal inversions such as *2Rb*. Overall, linkage disequilibrium within *An. arabiensis* decayed very rapidly (within 200 bp) across all chromosomes. However, elevated linkage disequilibrium was observed within some inversions, suggesting that recombination is reduced in those regions. The overall low levels of linkage disequilibrium suggests that association studies in this taxon will be very challenging for all but variants of large effect, and will require large sample sizes.

Genome-wide association studies have become a widely used and successful approach for identifying the genetic basis of traits in humans and model plant and animal species (Hindorff *et al.* 2013). As the price of genome sequencing decreases and genomic resources become available for a wider range of taxa, it is becoming increasingly possible to apply these approaches to nonmodel species. Association mapping tests for an association between a set of genome-wide single-nucleotide polymorphisms (SNPs) and a specified phenotype, with the expectation that one or more of these SNPs may be linked to the causal variant(s). As such, the level of genetic diversity and linkage disequilibrium (LD) in a species’ genome greatly influences the suitability and power of this approach (Gordon and Finch 2005; Hall *et al.* 2010).

LD describes the nonrandom association of alleles at different loci within a population and is influenced by many factors, including recombination rates, mutation rates, breeding systems, demography, and selection, all of which vary greatly between species (Ardlie *et al.* 2002; Gaut and Long 2003; Lewis and Knight 2012). The length of LD in a genome is a critical parameter in association studies because different levels of LD greatly affect the threshold set to account for multiple testing. Specifically, in taxa with long LD [*e.g.*, >1 Mb in dog breeds (Boyko *et al.* 2010)], fewer markers are assayed and, thus, the threshold is less stringent. However, with long LD, mapping resolution is low, which can make it difficult to identify the precise location of the causal genetic variant(s) (Hall *et al.* 2010). It is possible to localize causal variants much more precisely (Hall *et al.* 2010) in taxa with short LD [*e.g.*, LD decays < 1 kb in maize (Gore *et al.* 2009; Tenaillon *et al.* 2001)]. However, with rapid linkage decay, many millions of SNPs must be assayed to achieve a high likelihood that one or more of the assayed markers is in linkage with the causal variant(s) (Hall *et al.* 2010), which would be expensive and difficult to achieve even with whole-genome sequencing. Moreover, the effective number of independent tests in a genome scan is much greater when LD is short, resulting in a more stringent threshold.

As a result of variation in LD between species, it is important to assess LD before conducting an association study. In addition to its variation between species, LD also has been shown to vary greatly across a genome, often as a result of variation in recombination rates. For example, recombination is suppressed, and therefore LD greater, within chromosomal inversions that are segments of the chromosome that have broken off, rotated 180 degrees, and reinserted into the chromosome (White *et al.* 2007a and b). Such genomic variation in LD must be taken into consideration during association analyses (Ersoz *et al.* 2009). Finally, it is important to test for evidence of population structure, which results in allele frequency differences between subpopulations. Unless controlled for, such population structure may cause spurious LD between unlinked markers, resulting in false associations and/or inflated true associations (Kang *et al.* 2008; Lewis and Knight 2012).

Malaria in sub-Saharan Africa is transmitted by many members of the *An. gambiae* s.l. species complex. Of these, *Anopheles gambiae* s.s. Giles has been shown to be the most important vector of malaria in many regions, and thus has been the main focus of malaria vector research. However, there is growing evidence that the sister species, *An. arabiensis* Patton, replaces *An. gambiae* s.s. as the dominant vector in many areas with high insecticide-treated net coverage (Derua *et al.* 2012; Russell *et al.* 2011). As such, there is an urgent need to improve our knowledge of the behavior, ecology, and genetics of this somewhat-understudied vector to prepare for future vector control strategies.

In contrast to *An. gambiae* s.s. which is an almost entirely anthropophilic, nocturnal and endophagous feeder, *An. arabiensis* has been shown to exhibit much more variation for these traits (Lyimo and Ferguson 2009; Lyimo *et al.* 2013; White *et al.* 1972). Moreover, variation in some of these traits has been shown to have fitness costs [*e.g.*, host choice (Lyimo *et al.* 2013)], indicating that they may be under genetic control. This has led to considerable interest in applying association mapping to understand the genetic basis of these as well as other traits of medical importance. Although successful in many model taxa, the suitability of such an approach for *An. arabiensis* is unknown. Genetic studies in *An. gambiae* s.s. have detected high levels of diversity and estimated LD to decay within <1−3 kb (Harris *et al.* 2010; Neafsey *et al.* 2010; Weetman *et al.* 2010). Moreover, *An. gambiae* s.s. has been shown to exhibit a complex population structure with two incipient species (Favia *et al.* 2001; Gentile *et al.* 2001), five chromosomal forms (Coluzzi *et al.* 1979, 1985), and a new cryptic subpopulation [Goundry (Riehle *et al.* 2011)] reported. Less information, however, is available for *An. arabiensis*. Here we conducted whole-genome sequencing on the vector *An. arabiensis* to assess genomic patterns of diversity, genetic differentiation, and LD.

## Materials and Methods

### Samples, DNA extraction, and library preparation

Adult female *An. gambiae* s.l. mosquitoes were collected by aspiration from the villages of Lupiro (-8.38000, 36.66912), Sagamaganga (-8.06781, 36.80207), and Minepa (-8.25700, 36.68163) in the Kilombero Valley, Tanzania in 2012, and Ourodoukoudje (9.09957, 13.72292) in Cameroon in 2005. Samples were preserved in 80% ethanol. The head and thorax of each specimen was subsequently dissected and used for DNA extraction, which was performed with the QIAGEN Biosprint 96 system using QIAGEN blood and tissue kits (QIAGEN, Valencia, CA). *Anopheles arabiensis* samples were distinguished from other *An. gambiae* s.l. species complex members with the Scott polymerase chain reaction assay (Scott *et al.* 1993) and their DNA content quantified using the Quibit 2.0 Fluorometer (Life technologies, Grand Island, NY). Selecting samples with >80 ng of DNA, we prepared 24 individually barcoded Illumina paired-end sequencing libraries with insert sizes of 320−400 bp by using NEXTflex Sequencing kits and barcodes (Bioo Scientific, Austin, TX); 20 from Tanzania and four from Cameroon. The 24 samples were submitted to the Beijing Genomics Institute at the University of California-Davis for 100-bp paired end sequencing using the Illumina HiSeq2000. All 24 samples were sequenced at low coverage, with 12 samples per lane. In addition, three samples (2012LUPI059; 2012MINE001; 2005OKJ042) were selected to be sequenced together at greater coverage in a single lane. These were used to create a reference of high confidence variant sites for subsequent SNP detection in the lower coverage samples.

### Inversions

A number of polymorphic inversions have been identified among the species of *An. gambiae* s.l. species complex through karyotype analysis of ovarian nurse cells. There was insufficient material to karyotype the samples that were sequenced in this study as the abdomen was dissected for blood meal analysis. Therefore we conducted karyotyping on a sample of 37 indoor resting *An. arabiensis* collected in 2011 from the village of Lupiro, Tanzania. Ovaries were extracted from half gravid females, fixed in Carnoy’s solution, and banding patterns examined using a phase contrast microscope (Hunt 1973). Inversions were scored following Toure (1998) and are referred to following standard convention; the ‘+’ symbol followed by the letter by which the inversion is known refers to the standard arrangement (*e.g.*, *2R+^b^*), whereas the inverted arrangement is indicated by the letter alone (*e.g.*, *2Rb*).

### Data processing and variant detection

At the time of analysis (January 2013), the only *An. arabiensis* genome available was a transcriptome sequence. Therefore, we used the *An. gambiae* s.s. genome build AgamP3_15 as our reference. The *An. gambiae* s.s. genome was derived from the PEST strain that was fixed for the standard arrangement for all inversions polymorphic in *An. gambiae* s.s.

Before the alignment and mapping of our sequences, we removed adaptor sequences using SCYTHE (https://github.com/vsbuffalo/scythe) and conducted quality trimming using SICKLE (quality score > 20; https://github.com/najoshi/sickle). Reads were then aligned to the reference genome using BWA 0.6.1 (Li and Durbin 2009). We ran BWA with default parameters as well as with adjusted parameters (*e.g.*, changing number of permitted gaps and maximum edit distance) but found the default parameters to be the most optimal with our dataset in terms of mapped reads. The *An. gambiae* s.s. genome includes a number of unmapped haplotype contigs; we excluded data aligned to these contigs. We marked duplicate reads using PICARD (http://picard.sourceforge.net) and realigned reads around indels with realigner target creator and indel realigner from the Genome Analysis Toolkit, GATK 2.4.3 (McKenna *et al.* 2010).

We detected variants through a two-step process. First, to identify a set of high confidence reference variant sites, we combined the three high coverage samples and called SNPs using Unified Genotyper in GATK. After excluding indels (which are difficult to call reliably in the absence of very high, >100×, coverage), and apparent multiallelic SNPs (which may arise from errors in aligning reads to the genome and must be removed for many analysis programs), we filtered SNPs as per the Broad Institute’s (2012) Genome Analysis Toolkit (*i.e.*, GATK) hard filter recommendations (QD < 2.0; MQ < 40; FS > 60; HaplotypeScore > 13; MQRankSum<-12.5; ReadPosRankSum<-8.0), in addition to a quality score of >30 and combined depth of coverage >30×. The remaining biallelic SNPs constituted our high confidence SNP sites. We then repeated this process on a combined file of the 24 low-coverage samples, with the exception that the quality score threshold was reduced to 4 (Broad Institute 2012), and depth of coverage filter removed. We then excluded any SNP from this low-coverage data set if: (1) the site was not called in all samples; (2) it was not present in the high-confidence reference SNP set; (3) if the minor allele was not detected in at least five individuals; and 4) any multi-allelic SNPs. The resulting SNP set derived from the 24 low-coverage samples was used in all subsequent analyses.

### Coverage, LD, and genetic diversity analyses

Coverage across chromosomes was calculated with the depth of coverage tool in GATK. We used PLINK 1.07 (Purcell *et al.* 2007; Purcell 2009) to calculate pairwise LD as the genotyping correlation coefficient, r^2^. This analysis was not affected by phasing ambiguities because we calculated r^2^ directly from the genotypes rather than phased data (*e.g.*, Boyko *et al.* 2010). However, it is noteworthy, that r^2^ estimates from genotype data will not be identical to haplotype frequencies but are typically very similar (Purcell 2009). VCFTOOLS was used to calculate nucleotide diversity (π), SNP density, and F_ST_ (Danecek *et al.* 2011). Sliding window analyses were computed using custom scripts written in the python programming language (www.python.org), except F_ST_, which was computed with a sliding window tool within VCFTOOLS. The software SNPEFF (Cingolani *et al.* 2012) was used to determine the SNP effects by chromosomal arm. Figures were created using GNUPLOT (www.gnuplot.info). For the sliding window plots, the approximate locations of the telomeres, centromeres, and known inversions were taken from coordinates derived from *An. gambiae* s.s. as detailed in vectorbase (www.vectorbase.org).

## Results

To assess nucleotide diversity, population structure and LD, in *An. arabiensis*, we conducted Illumina 100-bp paired end whole-genome sequencing on 24 samples at low coverage; 20 from three villages in Tanzania and four from a single village in Cameroon. In addition, three of these samples (one from Cameroon, two from Tanzania) were sequenced at greater coverage to create a reference of high-confidence SNP sites. The number of reads generated per sample varied between 8 and 19 million reads for the low-coverage sequencing and 47 and 62 million reads for the high coverage sequencing.

We aligned sequences to the *An. gambiae* s.s. genome. *An. arabiensis* and *An. gambiae* s.s. are closely related sister taxa that occasionally hybridize to produce fertile female offspring (Slotman *et al.* 2005) and have been shown to have relatively low divergence across much of the genome (Neafsey *et al.* 2010). Nonetheless, it is noteworthy, that *An. gambiae* s.s. and *An. arabiensis* differ by a number of inversion polymorphisms (Coluzzi *et al.* 2002). In particular, on the X chromosome *An. gambiae* is fixed for the *Xag* inversion (which is absent in *An. arabiensis*), whereas *An*. a*rabiensis* is fixed for *Xbcd* which represents a complex of three fixed autapomorphic compound inversion arrangements. Furthermore, the 2La inversion which is fixed for the inverted arrangement in *An. arabiensis* (*2La*) is polymorphic in *An. gambiae* s.s. (*2La/+^a^*) and fixed for the standard arrangement (*2L+^a^/+^a^*) in the *An. gambiae* s.s. strain that was sequenced and used as a reference here.

**Figure 1:**
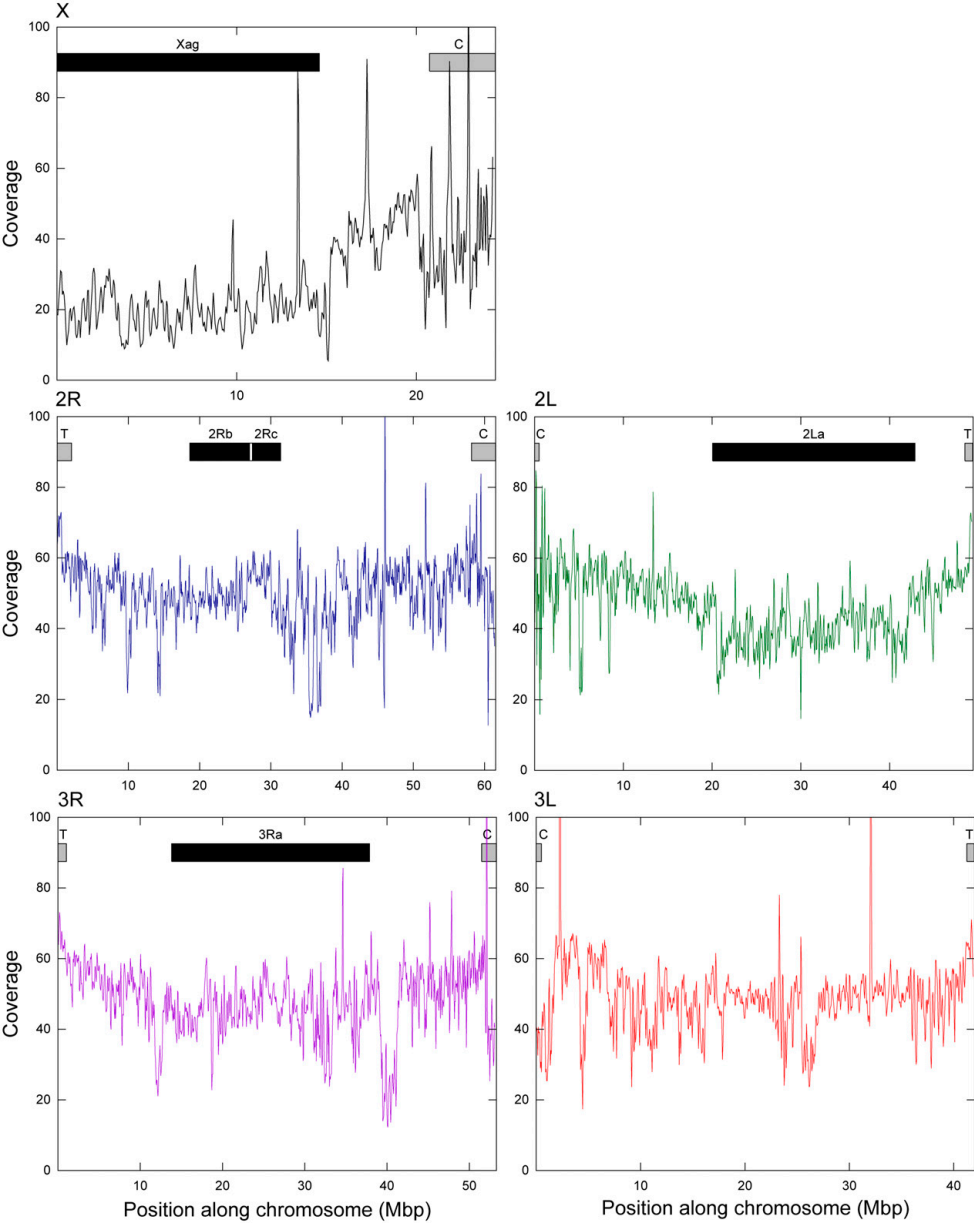
Sliding window analysis (bin 100 kb, step 50 kb) of combined coverage across the three high-coverage samples. Boxes depict approximate location of telomeric (T) and centromeric (C) regions (gray), and known inversions (black) based on *An. gambiae*.

Following alignment to the reference, we assessed coverage after excluding duplicate, low-quality, and poorly mapping reads. On the autosomes, coverage was high with 73–80% of bases exhibiting ≥30× combined coverage for the three high coverage samples, with 97–99% of the genome showing coverage overall (Table 1; Figure 1). On the X chromosome, coverage was lower, with only 38% of the genome exhibiting a combined coverage ≥30× for the three high coverage samples, and ~12% of the X chromosome showing no coverage (Table 1 and Supporting information, Figure S1). Similar findings were observed for the low-coverage samples (data not shown). Lower coverage on the X chromosome was particularly associated with the *Xag* inversion (Table 1, Figure 1, and Figure S1). Coverage also decreased proximal to the centromere on all chromosomes.

**Table 1:** Combined coverage and percentage of bases with 0 and ≥30× coverage across the three high-coverage samples per chromosomal arm

% Bases	X	2L	2R	3L	3R
0×	12.5	3.2	1.7	2.6	2.6
≥30×	38.3	73.8	80	77.8	77.5
Combined coverage	17,047,842	41,483,784	53,138,329	34,900,555	45,541,133

*An. arabiensis* is polymorphic for a number of inversions on the 2R and 3R chromosomes. To assess the inversions present in the Kilombero Valley of Tanzania we conducted karyotyping on ovarian nurse cells extracted from 37 blood fed *An. arabiensis* females collected from Lupiro in 2011, as ovarian nurse cells were not available for the Tanzanian samples sequenced here (see the section *Materials and Methods*). This revealed polymorphism for the *2Rb* inversion (*2R+^b^/+^b^* = 2, *2Rb/b* = 15, *2R+^b^/b* = 20), and *3Ra* inversions (*3R+^a^/+^a^* = 15, *3R+^a^/a* = 21) which is consistent with other studies of *An. arabiensis* in Tanzania (Petrarca *et al.* 2000).

### Genetic diversity

In total we identified 712,707 biallelic SNPs among our 24 low-coverage samples. This should be viewed as a conservative estimate because we applied a set of stringent filters to ensure a set of high-quality and high-confidence SNPs. Specifically, from the 2,574,223 SNPs remaining after standard quality filters were applied (see *Materials and Methods*), we excluded any SNPs that were: (1) not called in all 24 low-coverage samples (excluded 1,142,750 SNPs); (2) not present in the three high-coverage samples (excluded a further 317,549 SNPs); (3) where the nonreference allele was not observed in ≥5 samples (excluded a further 321,714 SNPs); and (4) any multiallelic SNPs, which includes SNPs biallelic within *An. arabiensis* but for two nonreference alleles (excluded a final 80,115 SNPs). Of the 712,707 SNPs in our final data set, we found these to be predominately in intergenic (34%) or intronic (21%) regions or ±5 kb of a gene (38%), with only 6% occurring in coding regions.

**Figure 2:**
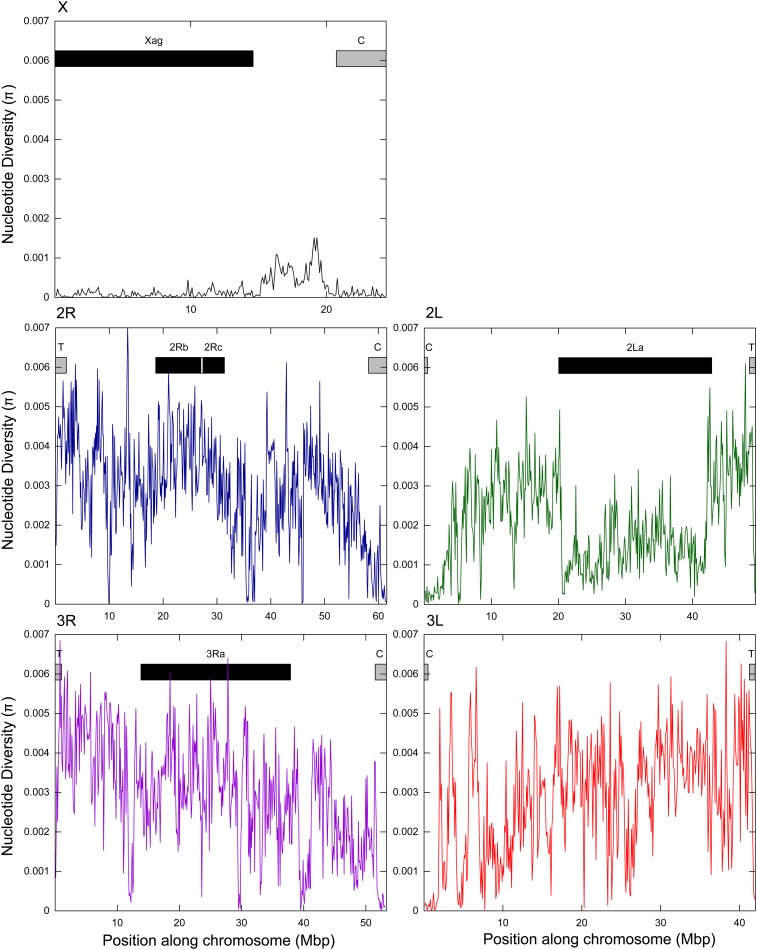
Sliding window analysis (bin 100 kb, step 100 kb) of diversity (π) by chromosome for the 24 low-coverage samples. Boxes depict approximate location of telomeric (T) and centromeric (C) regions (gray), and known inversions (black) based on *An. gambiae*.

In comparison with the autosomes, diversity was an order of magnitude lower on the X chromosome (X – density 1/2365 bp, π = 0.000213, autosomes − density ≥ 1/318 bp, π ≥0.00202; Table 2). Among the autosomes, SNP diversity was very similar for the 2R, 3L, and 3R arms (density = 1/221−1/227 bp, π = 0.00282−0.00294) but slightly lower on the 2L (density = 1/318 bp, π = 0.00202). Diversity was highly variable across chromosomal arms. On the X chromosome, diversity was lower in the telo/centromeric regions and within the *Xag* inversion, but higher outside of these regions (Figure 2). For the autosomes, lower diversity was associated with the centromeres as well as the *2La* inversion, but not the other inversions.

**Table 2:** Number of SNPs, SNP frequency, and nucleotide diversity (π) by chromosomal arm for 24 low-coverage samples

	X	2L	2R	3L	3R
No. SNPs	6308	126305	229666	150246	200380
SNP / × bases	2365	318	227	226	221
π	0.00021	0.00202	0.00293	0.00282	0.00294

SNP, single-nucleotide polymorphism.

### Population differentiation

**Figure 3:**
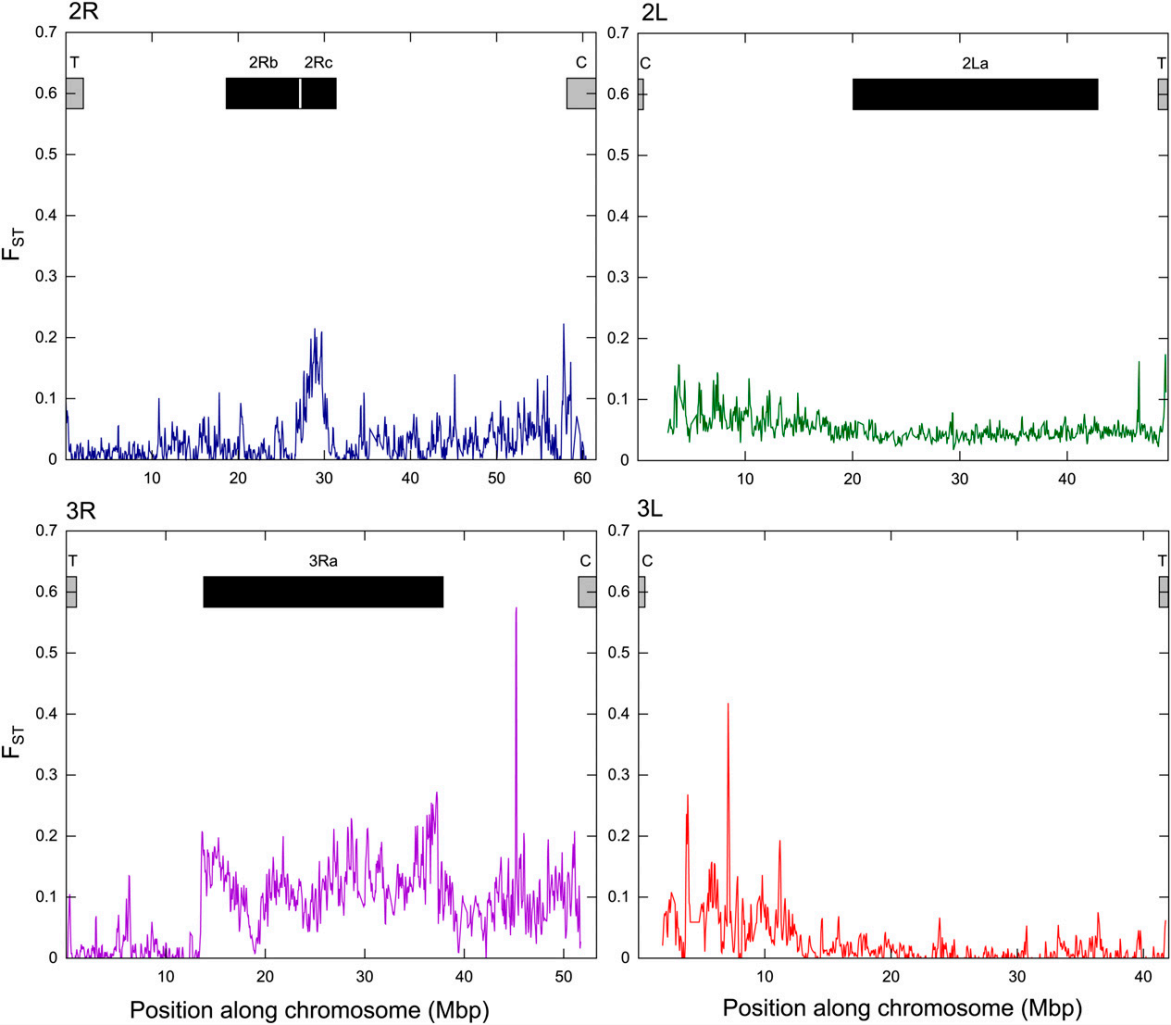
Sliding window analysis (bin 100 kb, step 50 kb) of F_ST_ plotted along the chromosome between the Cameroonian and Tanzanian samples. Windows with less than 100 values were excluded, which resulted in reduced representation in the centromeric regions, where SNP density was lower. Boxes depict approximate location of telomeric (T) and centromeric (C) regions (gray), and known inversions (black) based on *An. gambiae*.

Our set of genome wide SNP markers did not detect population structure among the three villages in Tanzania, which are located 14−57 km apart (F_ST_ = 0, Table S1). It is noteworthy though, that during filtering we excluded all multiallelic SNPs (see *Materials and Methods*), including SNPs where samples were biallelic for two non-reference alleles. As we had aligned our sequences to the *An. gambiae s.s*. genome, these biallelic nonreference SNPs were likely the most informative for identifying divergence within *An. arabiensis*. As such, we cannot exclude the possibility that some genetic sub-structuring exists between these locations, but on a finer scale than could be detected here with our conservative SNP dataset. Nonetheless, we did detect population structure between the Tanzania and Cameroon sites (F_ST_ = 0.057, Table S1), which is consistent with expectations for restricted gene flow between populations separated by great distances (>3000 km here, *e.g.*, Donnelly and Townson 2000). However, it is important also to note that the Tanzanian and Cameroonian samples were collected in different years (2005 and 2012), which means temporal genetic changes also may have contributed to apparent geographical differentiation. To identify the genomic regions contributing the most to divergence between the Tanzanian and Cameroonian samples, we estimated F_ST_ independently for each chromosomal arm. These analyses highlighted that divergence on the 3R (F_ST_ = 0.092−0.130) was higher in comparison to the other chromosome arms (F_ST_ = 0.016−0.051). Sliding window analyses showed that in contrast to other regions, divergence was elevated across most of the *3Ra* inversion (mean *3Ra* = 0.144), as well as on the *2Rc* inversion (mean *2Rc* F_ST_ = 0.131, Figure 3). An 8-Mb region up and downstream of the chromosome 3 centromere also showed elevated F_ST_ (Figure 3). SNP density was too low on the X chromosome for sliding window analysis.

### Linkage disequilibrium

**Figure 4:**
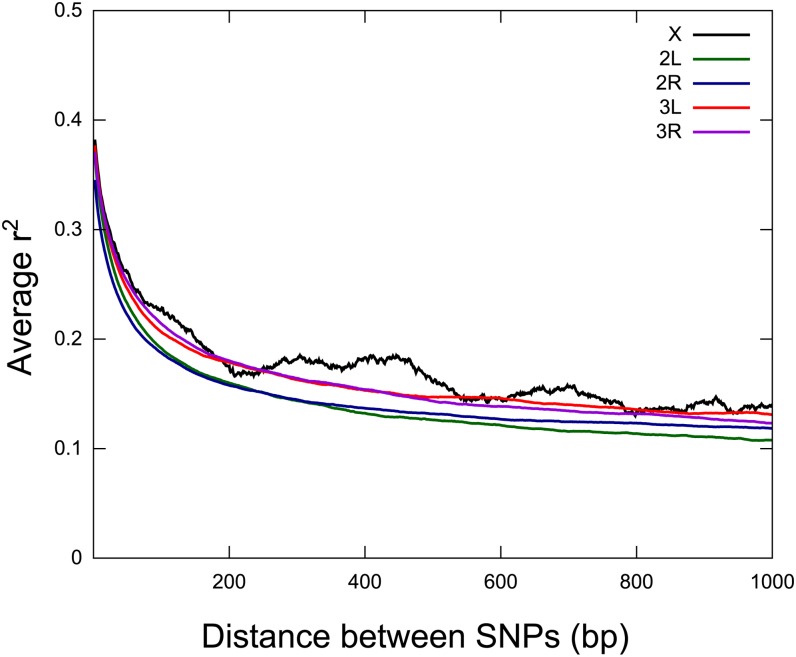
Decay of linkage (r^2^) by chromosome across the 20 Tanzanian samples.

We assessed linkage among the 20 Tanzanian samples. The four Cameroonian samples were excluded from this analysis to limit bias due to the confounding effects of population structure. The decrease in LD (r^2^) with physical distance for these samples is shown in Figure 4. These data show that average LD (r^2^) decayed rapidly to less than 0.2 within 200 bp in *An. arabiensis* (Figure 4), and this rate of decrease was very similar across all of the chromosomes.

**Figure 5:**
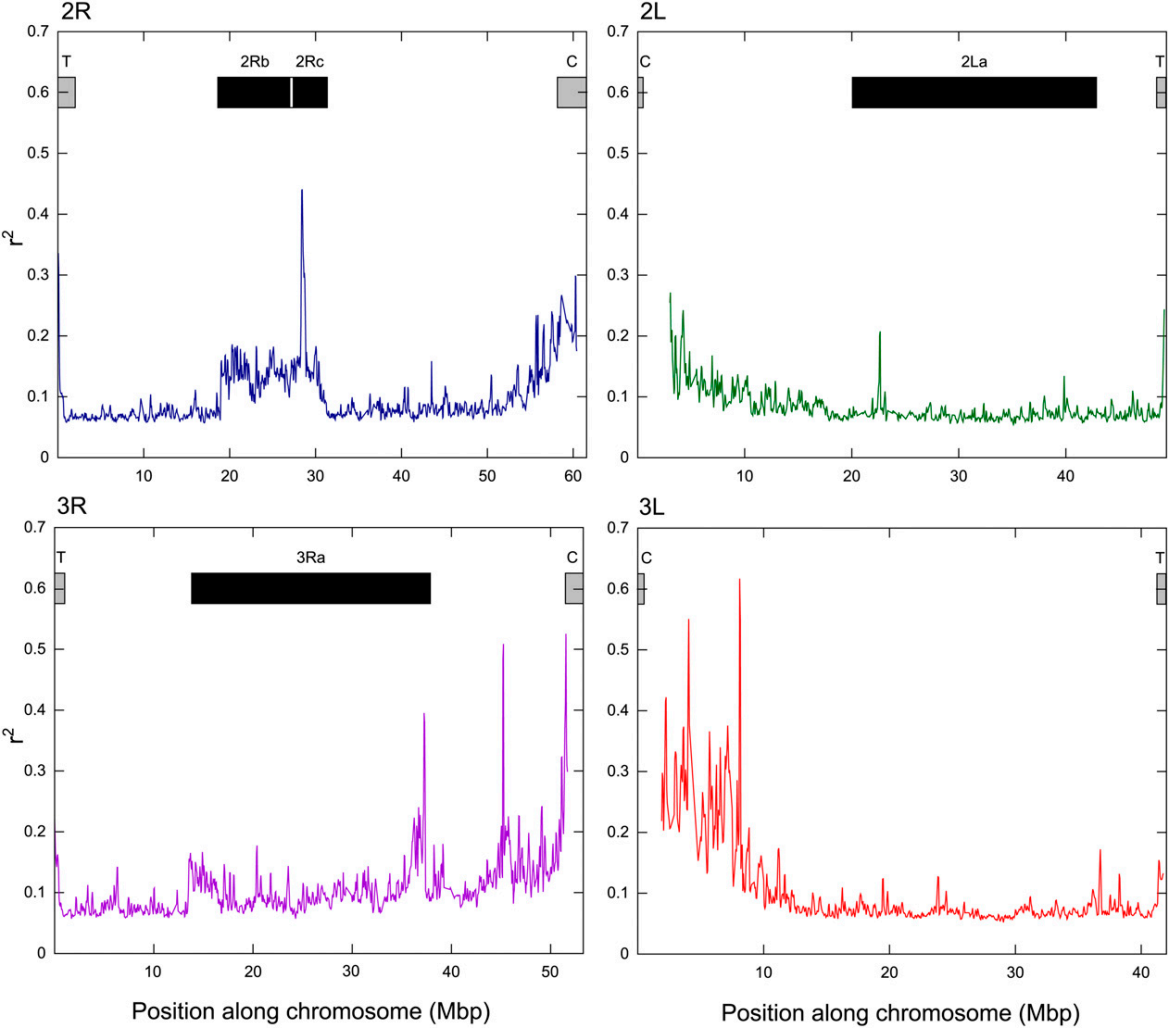
Sliding window analysis (bin 100 kb, step 50 kb) of linkage (r^2^) for SNPs separated by 1−10 kb in the 20 Tanzanian samples. Windows with less than 100 values were excluded, which resulted in reduced representation in the centromeric regions, where SNP density was lower.

To assess genomic patterns of LD, we plotted LD for all SNPs separated by 1−10 kbp by genomic location (Figure 5). This showed that although LD was generally low along the chromosome, it was increased around the centromeres and some inversions. Specifically, LD was elevated across the *2Rb* and *c* inversions and particularly at the proximal (nearer to centromere) *2Rb* breakpoint, as well as the proximal breakpoint of the *3Ra* inversion (Figure 5). Otherwise, elevated LD was limited to small sporadic regions. Elevated LD at the location of the *2Rc* inversion was unexpected because this inversion was not recorded in the small subset of samples from Lupiro in 2011 assessed here or in other studies from Tanzania (reviewed in, Petrarca *et al.* 2000). However, the number of samples and sites assessed in these studies was low, and therefore the inversion may have been missed. We did not find LD to be increased across the *2La* inversion, but this inversion is not polymorphic within *An. arabiensis* (only *An. gambiae* s.s.). SNP density on the X chromosome was too low for sliding window analyses.

## Discussion

### Genetic diversity

Consistent with expectations for an outbreeding species with large population sizes, we found high levels of polymorphism in *An. arabiensis*. Diversity estimates (Table 2) were slightly lower than reported for *An. gambiae* s.s. [density 1/35-250bp, **π**= 0.006−0.0208, Ensemble (Cohuet *et al.* 2008; Lawniczak *et al.* 2010; Morlais *et al.* 2004; Wilding *et al.* 2009)]. However, direct comparisons should be treated with caution, given the sampling differences between studies and the fact that the aforementioned estimates were derived from detailed studies of candidate genes or select loci (8−660) rather than low-coverage, whole-genome sequencing. Moreover, it is very likely that the stringent SNP calling thresholds we applied, combined with alignment to a sister genome, under estimated the true diversity levels in *An. arabiensis*.

Genomic diversity in *An. arabiensis* fluctuated across the genome; a pattern also seen in *An. gambiae* s.s. (Cheng *et al.* 2012; Cohuet *et al.* 2008; Holt *et al.* 2002; Wilding *et al.* 2009). Specifically, lower diversity was associated with the X chromosome, *2La* inversion, and telomeric and centromeric regions (Figure 2). The X chromosome plays a major role in postzygotic isolation between *An. gambiae* s.s. and *An. arabiensis* (Slotman *et al.* 2004, 2005), and is more diverged between these taxa than the autosomes (*e.g.*, Besansky *et al.* 2003; Cohuet *et al.* 2008; Neafsey *et al.* 2010). In particular, *An. gambiae* s.s. (reference genome) and *An. arabiensis* are fixed for alternative arrangements of the *Xag* inversion which covers ~60% of the X chromosome (Cohuet *et al.* 2008; Neafsey *et al.* 2010). Consequently, reduced X-chromosome diversity may reflect poorer alignment or mapping to the *An. gambiae* s.s. genome. However, reduced X diversity has also been shown in *An. gambiae* s.s. (Cohuet *et al.* 2008; Holt *et al.* 2002; Wilding *et al.* 2009), which indicates that additional biological factors, such as reduced introgression on the X chromosome, may be involved (Cohuet *et al.* 2008; Holt *et al.* 2002; Slotman *et al.* 2004, 2005).

Lower coverage attributable to mapping difficulties likely also explains the reduced diversity in the centro/telo-meres, which are highly repetitive regions and the *2La* inversion, which exhibits high divergence (Figure 1 and Figure 2). Specifically, the *An. gambiae* s.s. reference was derived from specimens fixed for the standard arrangement (*2L+^a^/+^a^*), which is highly diverged from the inverted arrangement (*2La*) which is fixed in *An. arabiensis* (Cheng *et al.* 2012; Neafsey *et al.* 2010). Indeed, Cheng *et al.* (2012) reported lower levels of coverage in *An. gambiae* s.s. specimens with the inverted *2La* arrangement. Finally, reduced diversity in the *2La* inversion may relate to the fact that this inversion is fixed in *An. arabiensis*, whereas the other inversions which did not exhibit reduced diversity are polymorphic and thus gene flux may contribute to diversity between arrangements (Cheng *et al.* 2012).

### Population structure

Cryptic population structure can result in spurious associations if not accounted for (Kang *et al.* 2008). Complex population structure has been found within *An. gambiae* s.s. (*e.g.*, Coluzzi *et al.* 1985; Favia *et al.* 2001; Lanzaro *et al.* 1998; Riehle *et al.* 2011) but fewer data are available for *An. arabiensis* (*e.g.*, Donnelly *et al.* 1999; Ng’habi *et al.* 2011). We found average F_ST_ between Tanzanian and Cameroonian *An. arabiensis* populations (F_ST_ = 0.057) to be lower than between Cameroonian populations of *An. gambiae* s.s. (F_ST_ =0.129, whole-genome sequence data, Cheng *et al.* 2012) which is consistent with suggestions that population structure is weaker in *An. arabiensis* than *An. gambiae* (*e.g.*, Kamau *et al.* 1999). Our findings are also consistent with studies showing little local population structure in *An. arabiensis* within East Africa (*e.g.*, Czeher *et al.* 2010; Lee *et al.* 2012; Nyanjom *et al.* 2003) but strong population structure at greater distances (*i.e.*, Cameroon and Tanzania) (Donnelly and Townson 2000). However, these data contrast with Ng’habi *et al.* (2011), who found significant F_ST_ values of 0.08−0.1 between villages within the Kilombero Valley Tanzania (20−50 km apart), and detected two subpopulations. Given that our studies did not assess the same sites (except Lupiro), this may reflect sampling. Alternatively, as mentioned previously, our stringent filtering of variants was biased toward removing the SNPs most likely able to detect fine scale structure. Assessments involving larger sample sizes and more study sites would be useful in clarifying local population structure.

Our analyses detected great variability in differentiation across the genome, with greater levels found on the 3R, particularly the *3Ra* inversion, as well as the *2Rc* inversion (Figure 3). Few studies have assessed inversions in *An. arabiensis* (Petrarca *et al.* 2000), but extensive research in *An. gambiae* s.s. indicates they are important for adaptation (*e.g.*, *2La* and *2Rb* with aridity) and are under strong selection (Cheng *et al.* 2012; Lee *et al.* 2009; Powell *et al.* 1999; Touré *et al.* 1998; White *et al.* 2007a). Moreover, partial reproductive isolation and niche differentiation has been detected between population subgroupings (chromosomal forms) with specific combinations of inversions (Coluzzi *et al.* 1977; Manoukis *et al.* 2008; Taylor *et al.* 2001). Our data suggest recombination and gene flow is restricted between standard and inverted arrangements of some inversions in *An. arabiensis*. Elevated divergence for specific inversions (*2La* F_ST_ = 0.247, *2Rb* F_ST_ = 0.149) among low overall genomic divergence (F_ST_ = 0.129) has also been reported in *An. gambiae* s.s. (Cheng *et al.* 2012) as well as Drosophila (*e.g.*, Corbett-Detig and Hartl 2012). The values here for the *An. arabiensis* inversions with elevated F_ST_ (mean F_ST_ *2Rc* = 0.13108, *3Ra* = 0.14345) were slightly lower than those reported for *An. gambiae* s.s. (above), but this could result from unbalanced representation of the inversions among our samples; something that we could not control or test for as our samples could not be karyotyped prior to sequencing (see *Materials and Methods*). If as suggested by our data, chromosomal inversions contribute to population structure in *An. arabiensis*, it will be critical for this to be accounted for in association studies particularly given the potential for the inversions to cosegregate with the trait of interest (Weetman *et al.* 2010).

### Linkage disequilibrium

The extent of LD in a genome is a key factor influencing the feasibility of association mapping studies (Lewis and Knight 2012). Overall, we found that LD decays rapidly within 200 bp in *An. arabiensis* (Figure 4), but we also detected elevated LD in regions of reduced recombination such as near centro/telo-meres (Figure 5). In particular, elevated LD extended ~8 Mb from the 3L centromere, as also observed in *A. gambiae s.s*. (~6 Mb, Weetman *et al.* 2010). Similarly, we observed elevated LD for the chromosomal inversions (Figure 5), which exhibit suppressed recombination due to a lack of crossing over in heterokaryotypes (Kirkpatrick 2010; Navarro *et al.* 1997). We found greater LD associated across the smaller *2Rb* and *2Rc* inversions and particularly at the proximal *2Rb* breakpoint, whereas LD was only elevated at the proximal breakpoint of the larger ~24Mb *3Ra* inversion (Figure 5). This pattern is indicative of gene flux, whereby double cross-over events or gene conversion results in recombination between arrangements near the center of large inversions, despite recombination rates near the breakpoints remaining near zero (Andolfatto *et al.* 2001; Navarro *et al.* 2000; Navarro *et al.* 1997).

The rapid decay of LD in *An. arabiensis* is broadly consistent with the short LD reported for *An. gambiae* s.s. immunity genes (<1 kb, Harris *et al.* 2010; Weetman *et al.* 2010) but is shorter than genome wide LD estimates of *An. gambiae* s.s. (<3 kbp, Neafsey *et al.* 2010). The difference with the latter study may be explained by the larger number and density of markers here, which allowed for greater resolution of LD in *An. arabiensis* than in *An. gambiae* s.s. (average spacing of SNPs ~3 kb, Neafsey *et al.* 2010). Such short LD contrasts with the LD structure of humans (>10 kb, Hinds *et al.* 2005; Pe’er *et al.* 2006), and many domesticated or selfing plants (~10 kb *Arabidopsis thaliana* and >100 kb in rice, Oryza sativa; Huang *et al.* 2010; Kim *et al.* 2007) but is consistent with findings for flies (*Drosophila melanogaster*, <30 bp; Mackay *et al.* 2012) and many outbreeding taxa for example the grapevine (300 bp, Vitis vinifera, Lijavetzky *et al.* 2007) and Norway spruce (100-bp Picea abies; Heuertz *et al.* 2006). Moreover, our results fit with expectations for wild species with high diversity, large outbreeding populations and recent population growth (Ardlie *et al.* 2002; Gaut and Long 2003; Lewis and Knight 2012), Nonetheless, the rapid LD decay presents considerable challenges for association mapping experiments as the number of markers and samples needed for there to be sufficient power to identify causal variants, would be prohibitive in most cases, except perhaps where causal variants are located in genomic regions with elevated LD (*e.g.*, regions with selective sweeps, Weetman *et al.* 2010). Similar limitations inhibited attempts to utilize association mapping in wild populations of Drosophila which also exhibit very rapid LD decay. In this case, resources such as the *Drosophila melanogaster* Genetic Reference Panel (Mackay *et al.* 2012) and Drosophila Synthetic Population Resource (King *et al.* 2012) have been produced in order to artificially create LD through the formation of a population of inbred recombinant lines that can be used for whole genome association mapping of traits. The future of association mapping in *An. arabiensis* likely rests on the production of a similar type of resource which could be used to dis-entangle the genetic basis of traits that can be phenotyped in a laboratory setting.

## Supplementary Material

Supporting Information
