# Supplementary material for: Diversity, Differentiation, and Linkage Disequilibrium: Prospects for Association Mapping in the Malaria Vector Anopheles arabiensis
Source: G3 (Bethesda). 2013 Nov 26;4(1):121–31. doi: 10.1534/g3.113.008326 (PMC3887528; doi:10.1534/g3.113.008326)
Supplement: Supporting Information [file supp_4_1_121__index.html]

Diversity, Differentiation, and Linkage Disequilibrium: Prospects for Association Mapping in the Malaria Vector Anopheles arabiensis — Supporting Information 

# Diversity, Differentiation, and Linkage Disequilibrium: Prospects for Association Mapping in the Malaria Vector *Anopheles arabiensis*

## Supporting Information for Marsden *et al.*, 2014

**Files in this Data Supplement:**

- Supporting Information - Figure S1 and Table S1 (PDF, 703 KB)
- Figure S1 - Sliding window analysis (bin 10kb, step 10kb) of the distribution of bases with zero coverage by chromosome for the three high coverage samples. (PDF, 555 KB)
- Table S1 - Average FST between populations. (PDF, 299 KB)
