# Supplementary material for: Diversity, Differentiation, and Linkage Disequilibrium: Prospects for Association Mapping in the Malaria Vector Anopheles arabiensis
Source: G3 (Bethesda). 2013 Nov 26;4(1):121–31. doi: 10.1534/g3.113.008326 (PMC3887528; doi:10.1534/g3.113.008326)
Supplement: Supporting Information [file supp_g3.113.008326_008326SI.pdf]

G3·Genes | Genomes | Genetics

Diversity, differentiation and linkage disequilibrium: prospects for association mapping in the malaria vector, *Anopheles arabiensis*

Marsden C, D. [*], Lee, Y. [*], Kreppel, K. [§,†] Weakley, A. [*], Cornel A. [‡], Ferguson, H.M. [†], Eskin, E. [**], Lanzaro, G.C. [*]

[*]Vector Genetics Laboratory, Department of Pathology, Microbiology, and Immunology, School of Veterinary Medicine, University of California - Davis, Davis, CA 95616, USA
[§] Ifakara Health Institute, Off Mlabani Passage, P.O. Box 53, Ifakara, United republic of Tanzania
[†] Boyd Orr Centre for Population and Ecosystem Health, University of Glasgow, Glasgow, G12 8QQ, UK
[‡] Department of Entomology, University of California, Davis, CA 95616, USA
[**] Department of Computer Science, University of California Los Angeles, CA 90095, USA

Data repository: Data for this manuscript has been deposited to the sequencing read archive (SRP033000) and the open access vector database POPI (https://grassi2.ucdavis.edu/PopulationData/OpenProjects/AaGenome/).

DOI: 10.1534/g3.113.008326

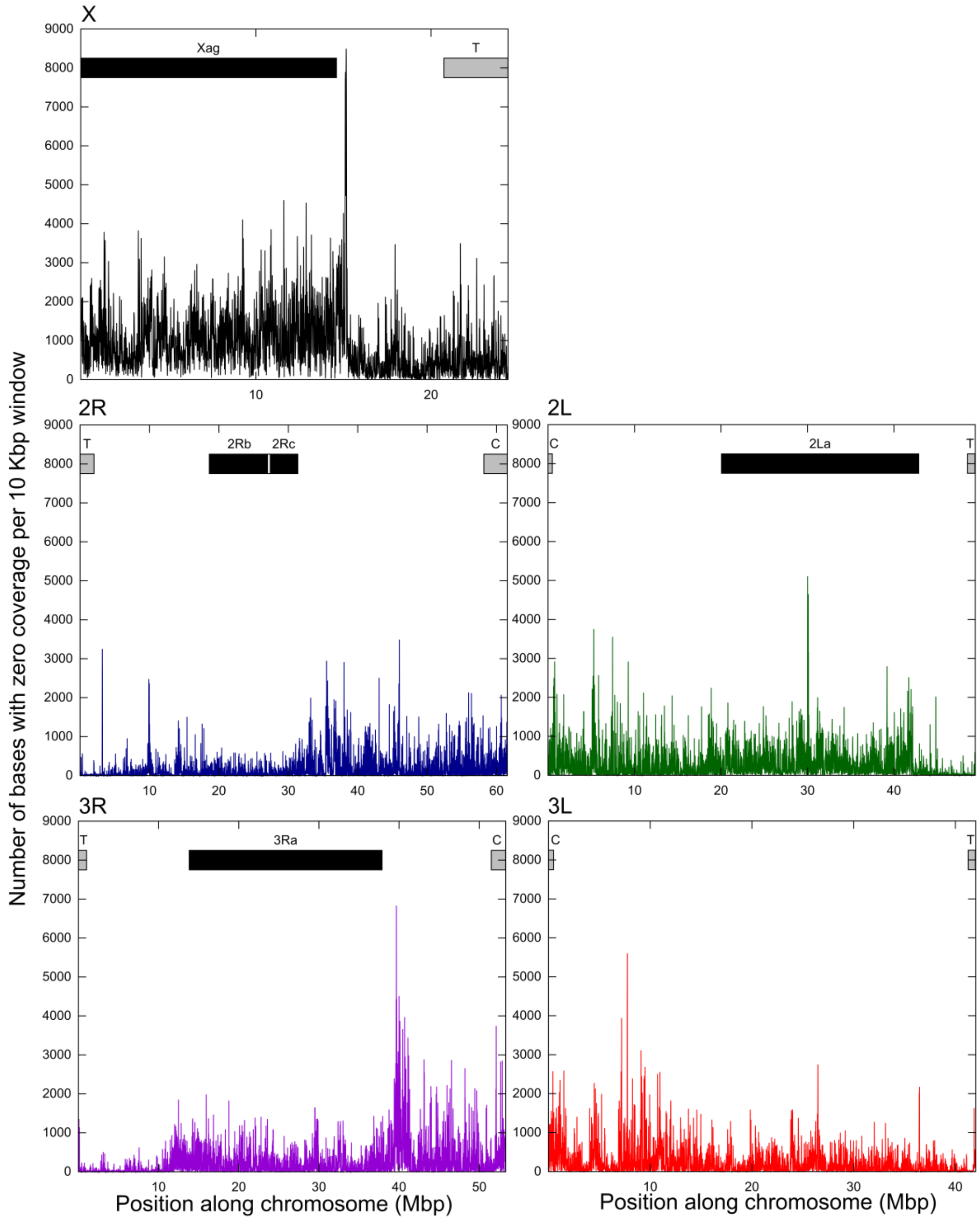

Figure S1 Sliding window analysis (bin 10kb, step 10kb) of the distribution of bases with zero coverage by chromosome for the three high coverage samples. Shaded areas depict locations of inversions. Boxes depict location of telomeric (T) and centromeric (C) regions (grey), and known inversions (black).

Table S1 Average F_{ST} between populations. Sample sizes are shown in brackets.

ALL	Okj	Lupi	Mine	Saga
**Okj(4)**				
**Lupi(8)**	0.054			
**Mine(4)**	0.060	-0.003		
**Saga(6)**	0.066	-0.004	-0.002	
			**Tanzania**	
**OKJ**			0.057	
