# Supplementary material for: Diversity, Differentiation, and Linkage Disequilibrium: Prospects for Association Mapping in the Malaria Vector Anopheles arabiensis
Source: G3 (Bethesda). 2013 Nov 26;4(1):121–31. doi: 10.1534/g3.113.008326 (PMC3887528; doi:10.1534/g3.113.008326)
Supplement: Supporting Information [file supp_g3.113.008326_FigureS1.pdf]

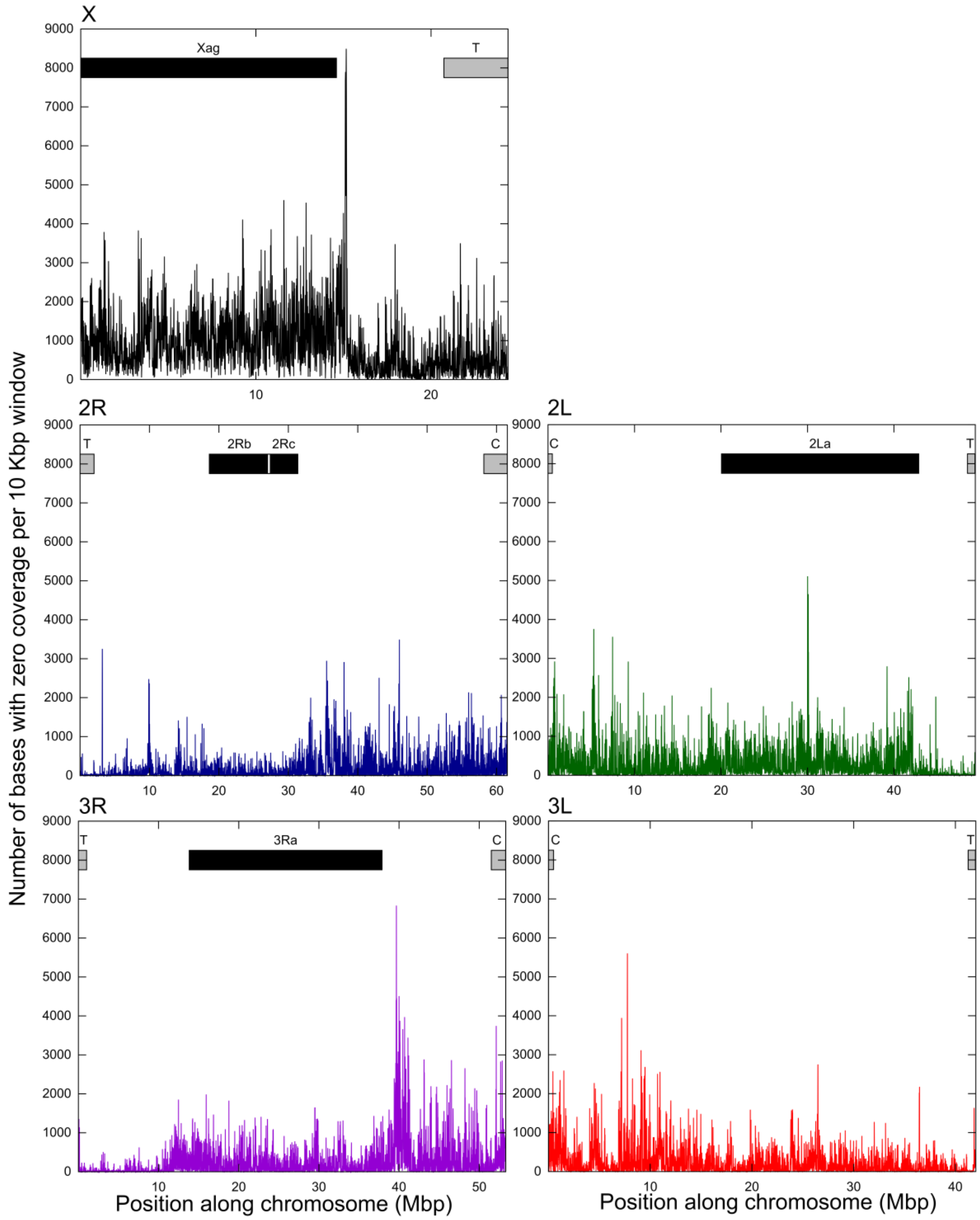

Figure S1 Sliding window analysis (bin 10kb, step 10kb) of the distribution of bases with zero coverage by chromosome for the three high coverage samples. Shaded areas depict locations of inversions. Boxes depict location of telomeric (T) and centromeric (C) regions (grey), and known inversions (black).
