# Supplementary material for: Diversity, Differentiation, and Linkage Disequilibrium: Prospects for Association Mapping in the Malaria Vector Anopheles arabiensis
Source: G3 (Bethesda). 2013 Nov 26;4(1):121–31. doi: 10.1534/g3.113.008326 (PMC3887528; doi:10.1534/g3.113.008326)
Supplement: Supporting Information [file supp_g3.113.008326_TableS1.pdf]

Table S1 Average F_{ST} between populations. Sample sizes are shown in brackets.

ALL	Okj	Lupi	Mine	Saga
**Okj(4)**				
**Lupi(8)**	0.054			
**Mine(4)**	0.060	-0.003		
**Saga(6)**	0.066	-0.004	-0.002	
			**Tanzania**	
**OKJ**			0.057	
